# Compliance with web content accessibility guidelines in ophthalmology social media posts

**DOI:** 10.1038/s41598-024-59838-2

**Published:** 2024-04-21

**Authors:** Meghan Sharma, Laura Huertas, Serena Shah, Alexandra Gil, Elena Bitrian, Ta C. Chang

**Affiliations:** 1 Bascom Palmer Eye Institute, University of Miami Miller School of Medicine, 900 NW 17th Street, Miami, FL 33136 USA; 2 Florida International University, 11200 SW 8th St, Miami, FL 33199 USA

**Keywords:** Social media, Visual impairment, Ophthalmology social media, Web accessibility, Web content accessibility guidelines, Americans with disabilities act, Quality of life, Outcomes research

## Abstract

This is a cross-sectional exploratory analysis of publicly available Internet data to examine compliance to web content accessibility guidelines (WCAG) on patient education social media posts in ophthalmology. WCAG ensures web content accessibility for those with disabilities (including visual impairment). A total of 100 social media posts were sampled from ten ophthalmology patient education social media pages and ten non-ophthalmology (cardiopulmonary) pages as the comparison group. Three independent graders evaluated the selected posts based on the WCAG 2 checklist by WebAIM, a non-profit affiliated with Utah State University, after its adaptation for social media posts. Validated accessibility standard labels: “0” for not meeting any standards, “1” or “A” for meeting bare minimum accessibility requirements, “2” or “AA” for meeting legal accessibility requirements, or “3” or “AAA” for exceeding accessibility requirements. There was not enough evidence to detect a difference in WCAG scores between ophthalmology and non-ophthalmology posts (*p* = 0.80). Forty-nine percent of scores for ophthalmology social media posts showed no compliance with any WCAG. The most common reasons that ophthalmology posts failed to meet criteria were due to color and contrast issues (39%). Most ophthalmology social media posts had low WCAG scores, indicating poor compliance to WCAG. Because social media is highly visual, reduced compliance to WCAG may create barriers for low vision individuals to successfully access patient education social media content.

## Introduction

Social media is used by more than 4.2 billion people worldwide^1^. In the ophthalmology community, social media is utilized mostly for patient education purposes^2^. Prior studies have shown that Internet use is not impeded by visual impairment, which supports the notion that some or many consumers of ophthalmology-based patient education social media content may be visually impaired^3,4^. Despite these findings, no study has analyzed compliance of social media posts intended for a visually impaired audience to web-based accessibility guidelines.

National guidelines have been published to increase accessibility for individuals with disabilities, including visual impairment. In 1990, the Americans with Disabilities Act (ADA) was passed to ensure “equal opportunity for persons with disabilities in employment, state, and local government services, public accommodations, commercial facilities, and transportation”^5^. Following the creation of the ADA, the Web Accessibility Initiative created the web content accessibility guidelines (WCAG) to enforce universal standards pertinent to web accessibility based on ADA objectives^6,7^. WCAG 2.0, a more recent version of WCAG, is divided into three tiers of recommendations: level A reflects minimum accessibility, level AA signifies redress of the most common issues to meet legal requirements, and AAA refers to the elimination of all obstacles to exceed requirements^8^. These guidelines have been recommended by the United Nations to guarantee web-based accessibility for all^9^. In 2018, WCAG 2.1 was developed to address mobile accessibility and people with low vision, cognitive disabilities, and learning impairments^5^. Two years later, WebAIM, a non-profit affiliated with Utah State University, created the WebAIM WCAG 2 checklist to condense the official WCAG 2.1 specifications^7^. This exploratory study investigates compliance to WCAG 2 among ophthalmology versus non-ophthalmology patient education social media posts using an adapted version of the WebAIM WCAG 2 checklist.

## Methods

This study does not involve human subjects and involves freely available web contents in the public domain, hence an evaluation and approval by the Institutional Review Board were not required per institutional policy. Researchers performed a web-based analysis using Instagram because it is a more visual platform than other social media sites and has the highest user base among social media platform when excluding those with strictly video-based content^10,11^. A 2023 systematic review evaluating the use of social media in plastic surgery found that Instagram was the platform with the highest engagement in the majority of studies and that Instagram could be an ideal social media marketing platform for patients 17–70 years of age^12^.

As an exploratory study, the present study evaluated online educational posts from ten ophthalmology and ten non-ophthalmology (cardiopulmonary) social media pages. Cardiopulmonary sites were chosen as the control group because researchers agreed that the intended audience of cardiopulmonary social media pages would not be enriched with visually impaired individuals^13^. The first ten public, English-language pages that appeared after searches for ophthalmology awareness pages and the first ten public, English-language cardiopulmonary pages were chosen after searches for the cardiopulmonary terms (Table 1). To find ophthalmology pages, search terms including “ophthalmology awareness,” “cataract awareness,” and “glaucoma awareness” were used. To find cardiopulmonary pages, search terms including “cardiopulmonary awareness,” “COPD awareness,” and “cholesterol awareness” were used. The account used for site identification and searching was an ophthalmology interest group account at a medical school. Briefly, the search algorithm of Instagram was outlined on the Instagram website. The algorithm uses the search text entered and ranks the resulting pages based on “popularity signals” (number of clicks, likes, shares and follows for a particular account, hashtag or place) while adjusting the order of the pages based on the user’s prior activities^14^. As an exploratory study, five posts from each site were systematically sampled (ie. every third post from the site in order to obtain a broader range of dates of posts, rather than just the first five posts) for review by three independent graders during April of 2023. The accounts chosen were not filtered based on the type of the account (e.g., government, academic) to simulate what a typical Instagram user may see when searching for posts on the site.

**Table 1:** Instagram accounts of posts sampled.

Ophthalmology education accounts	Cardiopulmonary education accounts
cureglaucomafoundation	copdhosa
glaucomaresearch	copd_awareness
laglaucoma	heart.health.nutritionist
eye_health_tips	lower.cholesterol.nutrition
nylasik	hearthealthmgmt
theglaucomafoundation	healthyhearties
carolinacataract	conquering_copd
dzeyemd	copdawarenesss
optimeyezinghealth	bloodpressuresolution
forglaucomasupport	pulmonary_disease_awareness

*NY* New York, *LASIK* laser-assisted in situ keratomileusis, *COPD* Chronic obstructive pulmonary disease, *HOSA* future health professionals organization, *MGMT* management.

All three graders had received at least an undergraduate college education and had previous exposure to the Instagram platform but were not ophthalmologists. Independent graders evaluated all posts based on an adapted version of the WebAIM WCAG 2 checklist. Study graders obtained permission from WebAIM to adapt their original WCAG 2 checklist for the intention of analyzing social media posts. The resulting checklist is displayed in Table 2. This checklist contains only the criteria from the original checklist that are relevant to the analysis of social media posts. For example, several original criteria specify keyboard function requirements, but keyboard functionality is a feature controlled by Instagram rather than post-creators. Study creators concluded that this criterion and other criteria not applicable to singular social media posts should be omitted in the adapted WebAIM WCAG checklist that was used for this study^7^.

**Table 2:** WebAIM ^©^ WCAG 2 checklist adapted for evaluation of social media posts.

A—Bare minimum accessibility	
1.	Descriptive and informative post title
2.	Content is presented in simple layouts using tables or text labels, for example, and the reading and navigation order makes sense
3.	Colors are not the sole method of conveying content
4.	Images have appropriate, equivalent alternative text or captions OR videos have appropriate audio descriptions or captions
5.	Contrast between the background and foreground is at least 3:1
AA—Accessibility Meeting Legal Requirements	
6.	Contrast ratio is at least 4.5: 1 (unless it is large text at least 18pt or 14pt + bold which can have 3:1)
7.	If the same visual presentation can be made using text alone, an image is not used to present that text
8.	Avoid duplicating heading or label text unless the structure provides adequate differentiation between them
AAA—Exceeds accessibility requirements	
9.	Contrast ratio is at least 7:1 (or if 18pt or 14pt + bold, the contrast ratio is at least 4.5:1)
10.	If post is part of a sequence of posts, there is an indication of the current page location (eg. post 2 of 3)
11.	Individual sections of content are designated using section headings beyond providing an overall document structure
12.	Words that may be unfamiliar, ambiguous, or used in a very specific way are defined through adjacent text, a definition list, or glossary
13.	The meaning of an unfamiliar abbreviation is provided by expanding it the first time it is used
14.	A more understandable alternative is provided for content that is more advanced than can be reasonably read by a person with 9 years of primary education
15.	An included image does not convey content OR is used when the information cannot be presented with text alone
16.	IF AUDIO: Audio with speech has no or very low background noise so that speech is easily distinguished

*WCAG* Web content accessibility guidelines, *WebAIM*^*©*^ web accessibility in mind.

*Original WCAG requirements and classification of accessibility developed by World Wide Web Consortium (W3C) in December 2008: Caldwell B, Cooper M, Reid LG, Vanderheiden G, Chisholm W, Slatin J, White J. Information Technology. W3C web content accessibility guidelines (WCAG) 2.0 (2008).

**Table reproduced with permission from WebAIM^©^.

Graders scored each post as “0” for not meeting any standards, “1” for bare minimum accessibility (A), “2” for accessibility meeting legal requirements (AA), or “3” for exceeding accessibility requirements (AAA)^7^. Each grader began with a dichotomous evaluation of the post using A criteria, wherein failure to meet all criteria merited a zero and success in meeting all criteria merited continuance to the AA category. Then, failure to meet all AA criteria merited a 1 and success in meeting all criteria merited continuance to the AAA category. Finally, failure to meet all AAA criteria merited a 2, and success in meeting all criteria merited a 3. To determine the posts’ color contrasts (necessary to assess A, AA, and AAA criteria), each grader input the foreground and background colors into a WebAIM contrast checker. To determine the posts’ readability (necessary to assess the AAA criteria), each grader input the written content into a Flesch-Kincaid Grade Level calculator. The Flesch–Kincaid Reading Grade Level formula is commonly used by researchers to determine if health information is at a reading level that is appropriate for patients by measuring semantic and syntactic difficulty^15^. For each post, graders recorded which criteria of the adapted checklist were unmet, if any. Graders were not masked to group assignment.

An ordinal logistic regression was conducted to assess the relationship between WCAG score (0, A, AA, or AAA) and source of social media post (ophthalmology or non-ophthalmology site). To keep observations independent, the regression model used the lowest of the three scores received by each post as its dependent variable. The lowest score was chosen to avoid overestimating any potential associations. Descriptive statistics based on all the scores and the lowest score were applied to the variables of interest. Additionally, Fleiss’s Kappa was calculated to determine the inter-rater reliability among all three independent graders.

## Results

**Figure 1:**
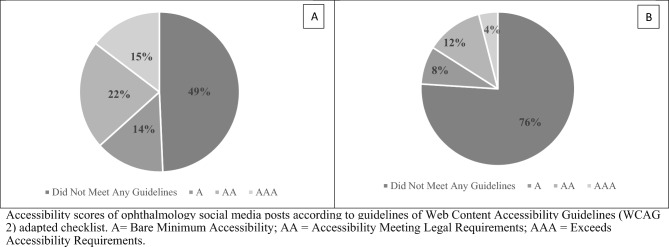
Overall scores and lowest scores for ophthalmology social media posts. Accessibility scores of ophthalmology social media posts according to guidelines of Web Content Accessibility Guidelines (WCAG 2) adapted checklist. *A* Bare minimum accessibility, *AA* accessibility meeting legal requirements, *AAA* exceeds accessibility requirements. (**A)** Distribution of all scores for 50 ophthalmology posts obtained from three independent graders (n = 150), (**B)** Distribution of lowest scores for 50 ophthalmology posts. The lowest score of the three scores obtained from three graders for each of the 50 ophthalmology posts was used (n = 50).

**Figure 2:**
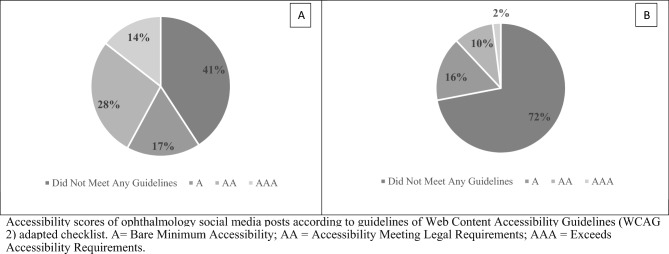
Overall scores and lowest scores for non-ophthalmology social media posts. Accessibility scores of ophthalmology social media posts according to guidelines of Web Content Accessibility Guidelines (WCAG 2) adapted checklist. *A* bare minimum accessibility; *AA* accessibility meeting legal requirements, *AAA* exceeds accessibility requirements. (**A)** Distribution of all scores for 50 non-ophthalmology posts obtained from three independent graders (n = 150), (**B)** Distribution of lowest scores for 50 non-ophthalmology posts. The lowest score of the three scores obtained from three graders for each of the 50 non-ophthalmology posts was used (n = 50).

**Figure 3:**
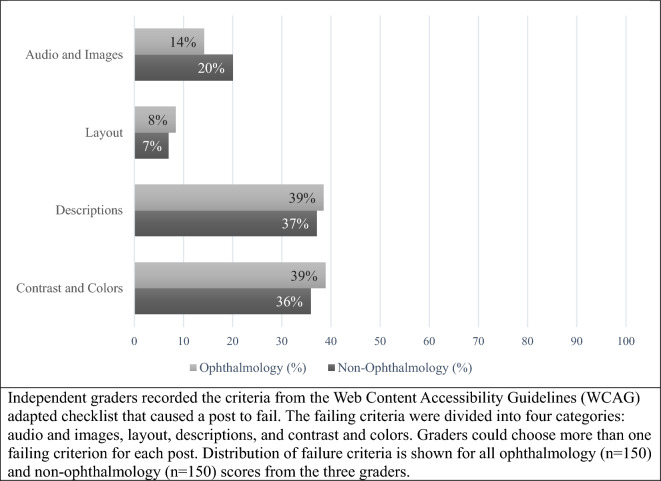
Failing criteria for ophthalmology and non-ophthalmology social media posts determined by three independent graders. Independent graders recorded the criteria from the web content accessibility guidelines (WCAG) adapted checklist that caused a post to fail. The failing criteria were divided into four categories: audio and images, layout, descriptions, and contrast and colors. Graders could choose more than one failing criterion for each post. Distribution of failure criteria is shown for all ophthalmology (n = 150) and non-ophthalmology (n = 150) scores from the three graders.

One hundred posts (50 ophthalmology and 50 non-ophthalmology posts) were reviewed by the three graders, resulting in a total of 300 scores (150 ophthalmology and 150 non-ophthalmology scores). Among them, the most common score received by both ophthalmology posts (49%, Fig. 1) and non-ophthalmology posts (41%, Fig. 2) was “0” for not meeting any WCAG standards. All reported reasons for why a post did not pass criteria are displayed in Fig. 3 and are expressed as four criteria categories: contrast and colors as criteria 3, 5, 6, and 9 from the checklist; descriptions as criteria 1, 12, 13, and 14; layout as criteria 2, 8, 10, and 11; and audio and images as criteria 4, 7, 15, 16, and 17. The most common category that ophthalmology posts failed was contrast and colors (39%) followed by descriptions (39%), audio and images (14%), and layout (8%). The most common category that non-ophthalmology posts failed was descriptions (37%), followed by contrast and colors (36%), audio and images (20%), and layout (7%).

**Figure 4:**
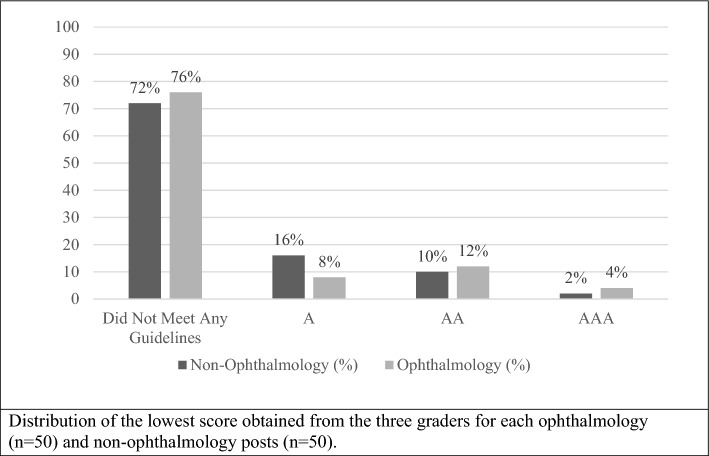
Lowest WCAG scores for non-ophthalmology and ophthalmology social media posts. Distribution of the lowest score obtained from the three graders for each ophthalmology (n = 50) and non-ophthalmology posts (n = 50).

The lowest score for ophthalmology posts was “0” for not meeting any guidelines for 76% of posts, A for 8% of posts, AA for 12% of posts, and AAA for 4% of posts. These results were compared to the lowest scores for non-ophthalmology posts, which were “0” for not meeting any guidelines for 72% of posts, A for 16% of posts, AA for 10% of posts, and AAA for 2% of posts (Fig. 4). By selecting the lowest score given to each post by the three graders, 100 scores (50 ophthalmology and 50 non-ophthalmology scores) were included in the ordinal logistic regression model. Based on the model, there was not enough evidence to conclude there was a difference in WCAG score between ophthalmology and non-ophthalmology social media posts (*p* = 0.80). However, there was only fair agreement among the scores given by the three graders (*κ* = 0.21, *p* < 0.0001), suggesting low inter-rater reliability.

## Discussion

Social media has played a growing role in patient education efforts^16^. Although many low vision individuals use social media, they likely encounter accessibility challenges. To the authors’ knowledge, this study is the first to analyze compliance to WCAG for ophthalmology social media posts and found no evidence to deduce that posts that were intended for a visually impaired audience adhered to more guidelines compared to those that were not. Our findings showed that most scores for ophthalmology social media posts did not meet any WCAG standards, which is consistent with other studies examining general WCAG compliance among web pages. A 2023 study by Utah State University tested the top one million web homepages and found that 96.3% were not compliant^17^. Another study found that the majority of e-government websites in India did not meet Level A standard with WCAG 2.1^18^. In our sample, more ophthalmology posts than non-ophthalmology posts did not adhere to any WCAG guidelines, with contrast issues being the most common reason for failure. Similarly, a study examining web accessibility of the library webpages of top-ranking United States post-secondary institutions found that approximately half of errors were categorized as contrast errors, directly impacted those with visual impairments^5^. Based on these findings, enhancing contrast either at the content creator or consumer’s level may significantly improve the accessibility and educational impact of these ophthalmology posts to the intended audience.

This overall poor WCAG compliance among ophthalmology posts may be explained by several factors. First, content creators may not be aware of WCAG standards. Despite the growing availability of accessibility information over the past two decades, website accessibility has not improved appreciably. An analysis by Swallow et al. contributed some of these findings to external factors, such as client and organizational attitudes to web accessibility or difficulty in enforcing accessibility legislation^19^. Moreover, creators may experience difficulties in utilizing the accessibility information provided by tools, guidelines, and resources. One study analyzing 17 students taking part in a web accessibility course concluded that WCAG is “far from testable for beginners” due to difficulty in comprehending the language used in guidelines^20^.

Tools such as the WebAIM WCAG 2 checklist were created to simplify the official WCAG 2.1 specifications into a more usable checklist for web creators; however, this study highlighted several limitations of the checklist. Many of the WebAIM criteria are subjective, such as “descriptive and informative post title,” which graders may evaluate differently. In our study, we attempted to decrease subjectivity and bias by having multiple graders assess posts; however, there was low inter-rater reliability, suggesting that scores of posts still varied despite using a controlled, stepwise evaluation algorithm. Additionally, the WebAIM WCAG 2 checklist may not be the most suited for content specifically addressing a visually impaired audience since the checklist aims to address a variety of disabilities. For instance, there are no criteria on the checklist referring to type of font used, as some fonts may appear more obscured or difficult to read than others. This may impact accessibility of the post for a visually impaired individual. Finally, the checklist was not the most suitable for analyzing social media posts, as many criteria from the original checklist needed to be omitted because they were not relevant for social media posts^7^.

This study has several limitations, including limitations with the search strategy used on Instagram to identify the sites used for analysis. Searches may vary based on factors such as previous activity of the account or popularity of a post, and this may cause variability in what searches are obtained^14^. Additionally, it is assumed, but not known, that visually impaired ophthalmological patients use Instagram and related posts for educational purposes, as opposed to validated sites supported by various professional organizations, such as the American Glaucoma Society or the American Academy of Ophthalmology. We had assumed that the content creators aimed to educate the public, while the main incentive for for-profit content creation is typically to exert and widen one’s digital influence. Hence, there may not be sufficient incentives for WCAG compliance as this may or may not increase the number of “views,” “shares,” or “likes” that signifies a post’s popularity. A future study could examine a comparison of WCAG compliance between official (e.g. government, professional and/or non-profit societies, academic institutions) and non-official sources (e.g., individual accounts). Future studies could also amend study design to see if WCAG compliance influences a post’s popularity, while a subsequent study may create and validate a new tool for analysis of social media posts to WCAG guidelines, since the original WebAIM WCAG checklist is tailored specifically to online websites. Moreover, many patients with visual impairment may use adaptive technology to access digital content. A future study could examine whether the use of specific devices meant for visually impaired individuals could enhance web content accessibility. Also, this cross-sectional study does not speak toward the overall trend of WCAG compliance, nor can we generalize on the variability of WCAG compliance using our modestly-sized sample for an exploratory study against the vast web content that currently exists on the Internet. Increasing the number of posts examined in a future study may provide greater insight to determine generalizability of the findings significance as this study was primarily an exploratory study focusing on a small number of sites and posts.

## Conclusion

Social media is an important tool in the dissemination of information for patients in ophthalmology. This study is the first to analyze compliance to WCAG for ophthalmology social media posts and found that there was overall poor compliance, potentially limiting the content accessibility for visually impaired consumers. Raising awareness in WCAG compliance can potentially improve ophthalmology patient educational access via social media for the intended audience.

## Data Availability

The datasets used and/or analyzed during the current study are available from the corresponding author on reasonable request.
